# Polymer-Based Scaffolds for Soft-Tissue Engineering

**DOI:** 10.3390/polym12071566

**Published:** 2020-07-15

**Authors:** Victor Perez-Puyana, Mercedes Jiménez-Rosado, Alberto Romero, Antonio Guerrero

**Affiliations:** 1 Departamento de Ingeniería Química, Facultad de Química, Universidad de Sevilla, 41012 Sevilla, Spain; vperez11@us.es; 2 Departamento de Ingeniería Química, Escuela Politécnica Superior, Universidad de Sevilla, 41011 Sevilla, Spain; mjimenez42@us.es

**Keywords:** freeze-drying, electrospinning, 3D printing, bioactive scaffolds, tissue engineering

## Abstract

Biomaterials have been used since ancient times. However, it was not until the late 1960s when their development prospered, increasing the research on them. In recent years, the study of biomaterials has focused mainly on tissue regeneration, requiring a biomaterial that can support cells during their growth and fulfill the function of the replaced tissue until its regeneration. These materials, called scaffolds, have been developed with a wide variety of materials and processes, with the polymer ones being the most advanced. For this reason, the need arises for a review that compiles the techniques most used in the development of polymer-based scaffolds. This review has focused on three of the most used techniques: freeze-drying, electrospinning and 3D printing, focusing on current and future trends. In addition, the advantages and disadvantages of each of them have been compared.

## 1. Introduction

A biomaterial is defined as any material used to produce devices that can replace a part or function of an organism in a safe, economical and physically plausible way; although it can also be referred as that material of biological origin or obtained from biological materials [1,2]. Nevertheless, the most accepted definition was given by William in 1987 as “a non-viable material used in a biomedical device intended to interact with biological systems” [3].

Nowadays, applications for the use of biomaterials include aesthetics, trauma damage and congenital or degenerative diseases, with biomaterials becoming a necessity since human beings want to live longer and better [4]. Currently the average life expectancy in the world is greater than 73 years; it is estimated that more than 900 million people are over 60 years old and by the end of the 21st century, according to the United Nations Organization (UNO), this digit will triple [5]. The search for improvements in the quality of life and the increase in life expectancy has made it possible for modern society to witness scientific and technological progress in various fields in recent years.

The modern field of “biomaterials” is relatively new to be able to compile its history. The first great advances took place at the beginning of the 20th century with the first vanadium steel alloys for fracture fixation or threads capable of being absorbed and degraded by the body. Since the 1960s, the implementation of protocols and statistical analysis of the results, the application of techniques to characterize the structure and surfaces of materials lead to an exponential development of the field of biomaterials with the first definition of biomaterial in 1974 and the creation of the Society for Biomaterials one year later. Since the 1970s, the cooperation of medicine with basic sciences (biology, physics and chemistry) and engineering has been the definitive impulse for the development of biomaterials [6].

Each biomaterial is defined by three factors: characteristics (composition and structure), properties (response of the material in real working conditions) and behavior (response to changes in the environment that surrounds it). The introduction of implants in the human body can activate the immune system, hence the importance of the concept of biocompatibility. Biocompatibility, which is the ability of a material to have an adequate response in a specific application (resistance to blood clotting, bacterial growth and normal healing) with an adequate response from the host (patient). Therefore, biomaterials must be efficient devices that do not generate disorder in the metastable process of the living being or the performance of the device in the inserted environment for an indeterminate period [7].

Biomaterials can be classified in different ways according to the response of the tissue or body to the implant: in case the tissues die, the material is considered toxic, if it forms fibrous tissue, the material is considered inert, if it promotes a link interface, it is considered bioactive and, finally, if it promotes tissue replacement, the material is considered soluble. At the beginning of the use of biomaterials in a more systematic way, in the 1950s, the search focused on bio-inert materials (1st generation). Over time, the search turned to the bioactivity of materials (2nd generation), and more recently, the focus has been on regeneration of true functional tissue (3rd generation), focusing more on the body’s response than the biomaterial’s response, which is to perform the function for which it has been designed with the minimum biological response of the patient. Efforts were devoted to increasing the life of the implant through its interaction with the host tissue interface, then the focus was on the development of biodegradable materials capable of being incorporated or absorbed (after dissolution) by the host tissue, and lately, the concept of biomimetics (4th generation), searching for materials that actively participate in the recovery process, working on the tissue in a specific way, with stimulation at the cell level [8,9].

Biomaterials can also be classified according to their duration, being provisional (temporary use) or definitive (permanent use), as well as their mechanical properties that make them act as hard tissue or soft tissue. However, the best known and most widely used classification, nowadays, is the one that classifies biomaterials according to their origin, as they can be natural or artificial. The latter, depending on the nature of the material, can in turn be classified as metallic, ceramic, polymeric and composite materials [10].

Tissue engineering is an interdisciplinary field that fundamentally requires cultured cell technology. Thus, it utilizes growth factors which allow the precise and continuous control of cell growth conditions and materials engineering for the development of scaffolds (biomaterials) that can imitate the structure of an organ [11,12]. A scaffold is a matrix whose main function is to serve as an anchoring platform for the adhesion of cells and, thus, allow their growth and proliferation, give rigidity to the implanted tissue and provide an empty volume for vascularization [13,14]. However, it must also fulfill other functions such as transporting, storing and releasing active factors, as well as stimulating specific cellular responses, contributing to the structural and mechanical integrity of the treated region. Therefore, characteristics such as external geometry, surface topographic characteristics (roughness or hydrophilic-hydrophobic character) and the scaffold microstructure (pore size, porosity and pores interconnectivity) influence cell-scaffold interactions. Furthermore, the biocompatibility, degradation and mechanical properties of the scaffold play a key role since they affect both the formation of tissue in vitro and its viability and functionality once inserted. Scaffolds must maintain the physical integrity and stability necessary to support the sterilization process and the ability to be stored for a long period of time. Lately, efforts have been made to evaluate the effect of the chemical and structural characteristics of scaffolds on cellular behaviors, that is, on cell adhesion, proliferation, migration and differentiation [15,16].

A suitable raw material to obtain scaffolds with an optimal internal structure has a positive effect on the activity of cells, so it is evident that the selection of a suitable raw material is crucial for an optimal cell growth. In this way, scaffolds can be either natural or synthetic, but it is essential that they exhibit their specific properties throughout the entire runtime [17,18,19]. Among the most used materials we find metallic materials, ceramic materials or polymers. This last type is probably the most versatile group of biomaterials used in the fabrication of biomedical devices. This type of material allows scaffolds to be processed with adequate control of the architectural parameters such as pore size and shape, porosity and pore interconnectivity, wall morphology, and surface area which are key issues for cell seeding, migration, growth, mass transport and tissue formation. The main drawback is the lack of hydrophilicity for cell adhesion, as well as its limited biocompatibility [20,21].

In general, synthetic polymers are divided into two types as biodegradable and non-biodegradable materials. Certainly, the biodegradable ones present the greatest application in tissue engineering. The degradation process of biodegradable polymers is the absorption of water and hydrolysis, as well as the enzymatic cleavage of the polymer chain [22]. Among the different synthetic polymers, the most used are poly-α-hydroxy esters such as polylactic acid (PLA), polyglycolide (PGA) and polycaprolactone (PCL), among others [22,23,24,25,26,27,28,29]. On the other hand, natural polymers (biopolymers) are widely used in tissue engineering due to their structure and properties where they combine low antigenicity and inflammation with high affinity for water and adequate cytotoxic responses, biocompatibility, biodegradability, and muco-adhesiveness. Biocompatibility increases the influence of the cell on the scaffold to regenerate damaged tissue. However, the poor mechanical and structural properties of these polymers prevent their widespread use. The most popular natural polymers are polysaccharides and proteins. Regarding the proteins to be used in scaffolds, it is worth mentioning collagen or gelatin, elastin, fibrin and even silk fibroin. Among the polysaccharides, starch, alginate, chitosan and derivatives of hyaluronic acid stand out [20,30].

There are numerous fabrication techniques for the development of polymer-based scaffolds: the ones based on 3D printing, such as rapid prototyping [23,31,32]; solvent-based techniques, such as solvent casting [33] or phase separation [34]; and others that are more specific, such as self-assembly [35]. Among them, one of the most traditionally used techniques is based on the formation of highly porous scaffolds whose microstructure is formed by either nanofibers or interconnected pores [36,37]. However, in the recent years, emerging technologies have acquired relevance in the field due to the possibility to produce highly controlled scaffolds in terms of mechanical and morphological properties. Microfluidics and 3D printing techniques are examples of those emerging techniques that allows obtaining a defined geometry, high control on the uniformity in the pore size and a good interconnectivity [37,38,39,40,41,42,43,44].

Thus, the aim of this work is the description of different processing techniques to develop polymer-based scaffolds. Specifically, a comparison between the traditionally used technique to produce highly porous scaffolds (freeze-drying), the most versatile technique to produce polymer nanofibrous scaffolds from almost any polymer which can be solved or melt (electrospinning) and the most innovative technique to process either acellular or cell-laden scaffolds (3D printing) has been considered as the main topic of this review. Therefore, freeze-drying, electrospinning and 3D printing are described with a general overview of each process together with their current trends. Finally, a comparison of the evolution of the techniques and the future perspectives are included as conclusions.

## 2. Freeze-Drying

Although other techniques like 3D printing allow obtaining scaffolds with better structural properties, freeze-drying technique allows obtaining 3D porous scaffolds with a global porosity higher than 90% and pore sizes in the range between 20 and 400 µm [45]. It is based on the freezing of a polymer solution followed by the sublimation of the solvent, obtaining matrices with an interconnected porous microstructure.

**Figure 1:**
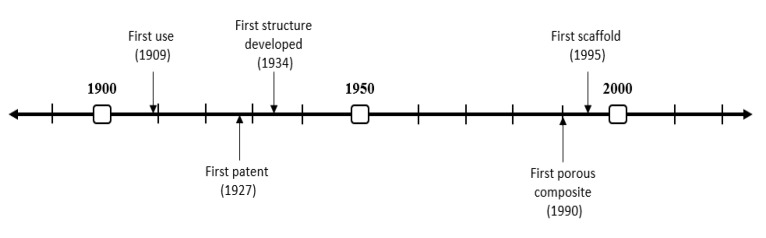
Timeline of the evolution of freeze-drying over history.

The evolution of the technique over the years can be seen in Figure 1. Its origin is found in 1909 when Shackell freeze-dried different biological materials. It is important to mention that it was not until 1927 when the first patent was registered by Tival. Later, in 1934, Flosdorf prepared the first stable structures via freeze-drying. However, it was in 1990 when De Groot et al. developed the first composite based on combinations of polyurethane and poly(l-lactic acid) (PU/PLLA) for tissue engineering [46]. Five years later, the first freeze-dried scaffold was produced [47]. Since then, the application of this technique in tissue engineering has grown exponentially [37,48].

### 2.1. Technique

This technique consists in the formation of a porous structure from a polymer solution by generating the conditions for its thermodynamic destabilization. The solution is separated into a polymer-rich phase and a polymer-depleted phase. The process typically starts with a cooling stage below the freezing point of the solvent, favoring the formation of crystals, until it is completely frozen. The second stage is carried out by means of a solvent removal process by freeze-drying. Thus, the polymer-rich phase tends to form the walls of the scaffold, while the polymer-depleted phase is the one that generates the pores in the internal structure of the acellular scaffold. The main advantage of this process is that the scaffold morphology (pore size and distribution) can be controlled by parameters such as the type of polymer, the concentration of polymer and the freezing temperature since they can regulate the nucleation mechanism and solvent crystal growth. Other additives, such as some surfactants or porogens, can contribute to improving morphological properties. High porosities and interconnectivity can be achieved with this technique, facilitating tissue development. However, the pore size that is usually achieved in this type of matrices is around the lower limit for its application in tissue engineering (TE). Furthermore, despite being a relatively simple technique of growing interest, its use is constrained to a small group of polymers, as well as to the lab-scale. The polymers that have been most used to prepare scaffolds using this technique have been synthetic polymers such as PLA, PGA, or poly(lactic-co-glycolic acid) (PLGA), as well as water-soluble biopolymers such as collagen and chitosan [49].

Considering the formation of hydrogels or sponge-like scaffolds, there are differing processing parameters to take into account. The most important parameters involving the formation of hydrogels are the gelation time, pH and the gelation temperature, among others. In contrast, the formation of sponge-like structures requires the control of other parameters such as the container used, the freezing temperature or cooling rate, and the pH of the solution or the solvent used.

### 2.2. Current Trends

Regarding current trends in the fabrication of scaffolds via the freeze-drying method, the main concern is to define the biomaterial microstructure in order to optimize cell adhesion, proliferation and differentiation. As commented before, the process has been developed in two different lines aimed at the formation of hydrogel-like (intermediate product) and sponge-like (final product) biomaterials. The use of this type of biomaterial is very complex due to its poor mechanical resistance that makes it difficult to apply it to bioreactors, which mechanically stimulate cell growth and proliferation. This problem is solvable in two different ways.

On the one hand, innovation in the field of hydrogels is found in the use of the nanoparticles that are included in the initial formulation to originate hydrogels. Among the different types of nanoparticles, magnetic nanoparticles that allow the stimulation of the biomaterial through the use of magnets (magnetic field) are especially interesting, thus avoiding the mechanical action that damages the structure of the material. In this way, a synergy occurs between the nanoparticles and the hydrogel, thus enhancing their use in regenerative medicine. The studies of Tanasa et al. (2020) and Shuai et al. (2020) follow this line since they are based on the development of scaffolds stimulated with either a magnetic field or a proper magnetic micro-environment [50,51]. The former produced silk-fibroin-based scaffolds with preosteoblasts imbibed, whereas the latter used the magnetic micro-environment for bone regeneration. On the other hand, nanoparticles with other objectives have also been studied, e.g., Aradmehr et al. (2020), in their work about the inclusion of silver nanoparticles in scaffolds based on a lignocellulosic film and a chitosan hydrogel [52]. Another example to point out is the study of Mokhtari et al. (2020), about the combination of gold nanoparticles with chitosan hydrogels in order to reinforce the structure and improve the cytocompatibility [53].

On the other hand, the structure of the sponge-like biomaterials has a high porosity (greater than 80%), making its control and definition essential for potential application in regenerative medicine (specifically in tissue engineering). To achieve it, current studies promote controlling the parameters of the freeze-drying process, specifically the cooling rate of the structure, as it will define the size of crystal formation (and, therefore, the pores of the final scaffold). In this sense, the study of Perez-Puyana et al. (2019) gave a general overview of the effect of each processing parameter on the properties of polymer-based sponge-like scaffolds [54]. Nevertheless, there are other studies that are based on the effect of a specific parameter. The most studied variable is the freezing temperature, highlighting the studies of Zamanian et al. (2014) or Reys et al. (2017) evaluating the effect of the freezing temperature on gelatin-based and chitosan-based scaffolds, respectively. Both studies obtained better properties for those scaffolds fabricated at a lower temperature [55,56]. On the other hand, Schwarzenbach et al. (2002) reported that the use of hydrophobic containers favors the elimination of aqueous solvents, improving the process [57].

## 3. Electrospinning

**Figure 2:**
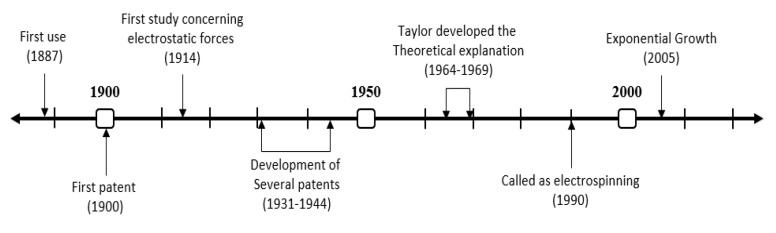
Timeline of the evolution of “electrospinning” over history.

The beginnings of electrospinning go back to the beginning of the 20th century, being patented between 1900 and 1902 by scientists Cooley and Morton (although in those years the technique was not known by the name that is used at the moment). Later, between 1931–1944, Firmhals got 22 patents on the mechanism [58]. However, it was not until 1964 that Taylor presented his theoretical explanation of the technique, and the known “Taylor’s cone” [59]. The technique was performed to produce materials with different applications as filters, although this did not receive its name until 1990 (Figure 2).

### 3.1. Technique

Electrospinning is a technique that allows the formation of continuous ultrafine membranes formed by polymeric fibers. The process consists of the application of a high voltage to induce the evaporation of the solvent of a polymeric solution, leading to the formation of micrometric and nanometric fibers on a metallic surface called the collector [60].

**Figure 3:**
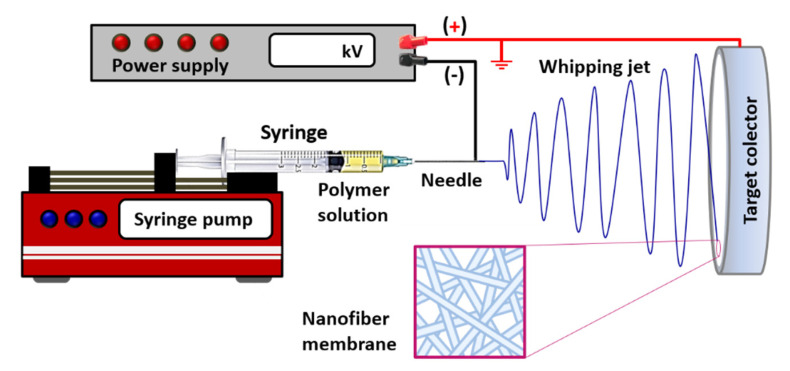
Schematic illustration of the electrospinning process and the fibrous structure achieved.

In this technique, a high-potential electric field (around 10–30 kV) is applied between a metallic syringe needle, through which a polymer solution emerges with a controlled flow rate, and a metal grounded collector (Figure 3). The high electrical potential generates strong repulsions on the surface of the polymer solution, which constitutes the main driving force of the process, producing, first, a deformation at the outlet of the capillary needle in the form of a conical droplet (Taylor cone), followed by a sudden projection of a liquid jet, from the tip of the cone in the direction of the collector.

Subsequently, bending instability of the jet takes place, causing the formation of large loops parallelly oriented to the collector in the whipping jet zone. This zone is the one that presents the greatest contribution to the generation of nanofibers, as it leads to a severe elongation that produces fiber thinning at stretching ratios in the order of thousands [61]. Simultaneously, the solvent evaporation occurs, giving rise to the solidification of polymeric fibers, which are continuously deposited on the target collector in that it forms a nanofiber membrane, with a typical random orientation. There is good interconnectivity between the pores of the membrane, although the pore sizes achieved are usually smaller than those obtained with other techniques.

The morphology of the membranes depends on many variables that can be grouped into three types: (i) properties of the solution (concentration and nature of the polymer, dielectric properties of the solvent, viscosity, conductivity and surface tension); (ii) processing parameters (voltage, flowrate, capillary dimensions, needle to collector distance and polarity); (iii) environmental factors (relative humidity and temperature).

Electrospinning technique allows to obtain fibers with diameters between 2 nm and several μm with a wide variety of either synthetic polymers or biopolymers [62]. The optimization of the above variables is essential to achieve more uniform fibers with diameters at the nanoscale. In general, it is a processing technique that has acquired great relevance, in a wide variety of applications and disciplines, including TE. Thus, this technique presents a great potential for the manufacture of functional artificial tissue, thanks to electrospun polymer matrices’ ability to mimic native extracellular matrix (ECM), through its micro and nanofiber structure with interconnected pores. The effect of each parameter on the morphology of the fibers is summarized in Table 1.

This technique manufactures acellular scaffolds. However, several studies are demonstrating the ability of these electrospun matrices to adhere to a wide variety of cell types, allowing their proliferation and tissue development. To date, studies have been conducted, in the field of TE, with scaffolds of electrospun matrices with functionality for the generation of epithelial or muscle tissues, bones, cartilages and blood vessels. The most commonly used synthetic polymers in tissue engineering, due to their good electrospinning behavior, their good ability to mimic ECM and their good cytocompatibility characteristics and biodegradability have been PLA, PGA and PCL or their copolymers (e.g., PLGA, PLLA or PCLL) [63,64,65]. Among naturally occurring polymers, the most commonly used in electrospinning processes for TE applications have been some proteins such as collagen (or gelatin), often in combination with another polymer (e.g., PCL), elastin and fibrinogen or silk fibroin. Some polysaccharides (chitosan, alginate or hyaluronic acid) have also been used, with excellent results in terms of morphological and functional properties [63,64,65,66,67].

All the progress achieved with the electrospinning process has been driven mainly by the great versatility of the process, by its low cost and by a great efficiency in obtaining membranes formed by micro and nanofibers with good pore interconnectivity. However, it also involves significant limitations, such as the time required to obtain suitable thickness membranes or problems to the cytotoxicity of solvent residues, the slack of polymer biocompatibility, or the smaller pore sizes obtained as compared to other techniques. These sizes may be suitable for some applications such as blood vessel generation, but they can result in some constrains for cell migration through the scaffold structure, as is the case for the development of bone or cartilaginous tissue.

### 3.2. Current Trends

In the last years, among the variety of polymers that can be used to obtain electrospun fiber mats, biopolymers (either protein or polysaccharide-based) are demanding more attention in biomedical applications. As more knowledge becomes available, it can be foreseen that these biopolymers will likely play an important role in the development of biomaterials with sustainability applications in tissue engineering.

The electrospinning technique is changing that, as with sponge-like biomaterials, implies a modification of its microstructure. Indeed, this modulation has been carried out by modifying the process conditions during the biomaterial formation process. Specifically, there are more and more studies that adjust the speed of rotation of the collector to achieve a certain orientation of the fibers, thus we control their microstructure and, therefore, two key aspects such as porosity and mechanical properties. On the other hand, coaxial electrospinning led to core–shell fibers and their ability to preserve the bioactivity of incorporated-sensitive biomolecules (such as drug, protein, and growth factor) and to subsequently control biomolecule release to the targeted microenvironments to achieve therapeutic effects. Such qualities are highly favorable for tissue engineering and drug delivery, and these features are not able to be offered by monolithic fibers [93]. Apart from that, melt electrospinning is another technique based on electrospinning, which allows the production of fibrous structures from polymer melts in a similar way to solution electrospinning and is applicable to different materials including ceramics [94].

However, there is a growing trend that combines the electrospinning technique with other techniques to fabricate a scaffold with combined properties. In this sense, in a similar way to the previous technique, electrospinning has also been combined with the nanoparticles field to drive up the properties of the membranes produced, e.g., the survey driven by Manjumeena et al. (2015) to obtain PVA fibers with gold nanoparticles with potential anticancer properties [95]. On the other hand, there are some studies devoted to the combination of electrospinning and 3D printing to produce a hybrid scaffold. Naghieh et al. (2017) presented pioneering research to create a composite based on the combination of poly(lactic acid), gelatin and forsterite [28]. In addition, Rajzer et al. (2018) combined gelatin and PLLA to produce scaffolds to promote nasal cartilages and subchondral bone reconstruction [96]. More recent is the work of Chen et al. (2020), in which the combination of these techniques was applied to cartilage regeneration [97].

Recently, electrospinning has been combined with another molecular printing technique allowing the modulation of the surface of the biomaterial by including small proteins and peptides, favoring surface-cell interaction. This technique is called molecular imprinting and is based on the study of surface-peptides interaction to promote cell adhesion and proliferation over the surface of the scaffold [98]. Especially relevant are the studies of Chronakis et al. (2006), in which the combination of electrospinning and molecular imprinting [99]. Other approaches were produced combining electrospun nanofibers and the molecular imprinting technique [100,101].

## 4. 3D Printing

**Figure 4:**
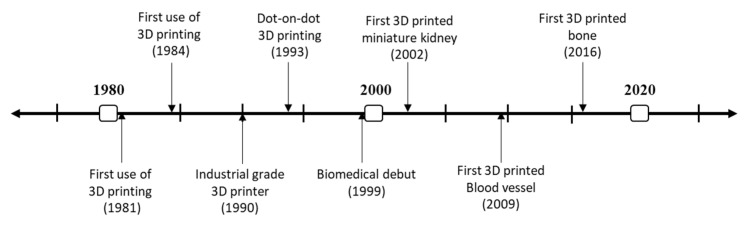
Timeline of the evolution of “3D printing” over history.

A timeline with the evolution of the 3D printing technique is shown in Figure 4. The first documented use of 3D printing dates back to 1981. Then, the first stereolithographic process was patented in 1984 by three French researchers [102]. However, the 1990s supposed the true growth of the technique with the development of the 3D printers of industrial grade in 1990, and 1993 was the year in which Solidscape developed their dot-on-dot 3D printing technique. This technique integrated the use of polymer-jet fabrication with high precision models, opening new application horizons for the technique like regenerative medicine [103]. The first biomedical use of 3D printing dates back to 1999, and since then, the number of studies relating 3D printing and biomedicine have shown an exponential growth. The first biomedical milestone was in 2002, when a miniature of a kidney with the same features was 3D printed. Other important dates to mention are 2009, when Organovo produced the first 3D printed blood vessel, and 2016 for the fabrication of the first 3D printed bone [104].

### 4.1. Technique

Most of today’s production of scaffolds for tissue engineering uses the conventional techniques described above. However, these techniques may impose some limitations on the design of the scaffold, providing only relative control over its morphology. Therefore, there is still a growing demand in TE for the development of techniques that allow to achieve a high degree of control over the morphological, biological and mechanical properties of scaffolds, with the aim of mimicking the macro and microstructure of the different types of tissues to be replaced or regenerated. Moreover, the progress in this field is favoring an increasingly specific demand, which converges towards the application of a personalized medicine in TE. Thus, several techniques have been recently developed that allow the manufacture of scaffolds with precise spatial control of their structure, from designs mimicking images of specific functional tissues.

There is still some confusion with the term 3D printing, which has been used either to describe a specific technique (e.g., 3D printing using polymeric powder as a raw material) or to encompass a set of them. This set of techniques has been denoted by different authors as rapid prototyping or as additive manufacturing 3D printing. It consists of a group of techniques that can directly generate structures, layer-by-layer, from models obtained using computer-aided design (CAD) techniques. Brief descriptions of the 3D printing technologies of greatest application potential in the field of tissue engineering are given as follows:

#### 4.1.1. Stereolithography (SLA) 3D Printing

This technique is basically used to manufacture solid 3D objects by consecutively printing thin liquid layers of UV curable material. After the addition of each layer, a spatially controlled photopolymerization stage is applied. The process is repeated layer by layer until the 3D scaffold is fully formed. The uncured polymer is then removed, keeping intact the structure of the designed model, which undergoes a new curing stage to reinforce the 3D structure. The resolution obtained with this technique is higher than others of additive manufacturing. Moreover, the fact that the resin used is liquid facilitates the removal of the excess material, although it also imposes certain restrictions on the design of the stereolithography equipment. However, the biggest challenge for its application to TE is the difficulty of finding suitable reactive resins that do not present cytotoxicity problems [105]. A variant of this technique, which uses light photopolymerization is digital light processing (DLP).

#### 4.1.2. Fused Deposition Modeling (FDM)

**Figure 5:**
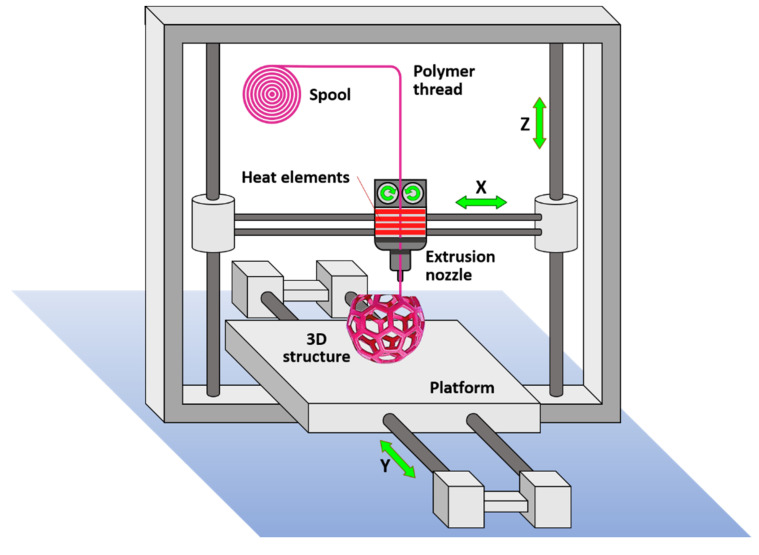
Schematic illustration of 3D printing by the modeled fused deposition technology.

FDM is a 3D printing technology based on the extrusion of molten polymers. As illustrated in Figure 5, the polymer thread is loaded from a spool and fed to a extrusion head where it is heated over the melt temperature, flowing through an extrusion nozzle that moves according to spatial coordinates that are specified by the CAD model, for each cross-sectional layer. The process repeats layer by layer until the 3D object is formed. The required mechanical integrity is achieved by fusing the consecutive layers after deposition. The technique allows the use of multiple nozzles that allow the use of established composition gradients in the three dimensions of the object, which can be used to tune its mechanical properties. In the FDM 3D printing, the resolution and dimensional accuracy is moderate as compared to other 3D printing technologies and strongly depends on the rheological properties of the thermoplastic polymer. This is a major limitation in the selection of the polymer that can be used in TE, especially for the use of biopolymers or biodegradable polymers [106]. FDM is among the most cost-efficient technologies to produce scaffolds for TE, however it typically leads to regular and simple porous structures.

#### 4.1.3. Selective Laser Sintering (SLS)

SLS is a 3D printing technology in which a very thin layer of dust is deposited with the help of a coating blade or a roller, forming a bed of a few tenths of a millimeter, in a vat that has been heated to a temperature slightly below the melting point of the dust. Sintering occurs at specific locations, after scanning the surface of each layer, by means of a high-powered laser that generates local heat at the points indicated by the CAD model for each layer. The process is consecutively repeated layer by layer. The use of the laser allows the application of this technique for different types of materials (polymeric, metallic or ceramic). The use of composite materials (e.g., PLLA and carbonated hydroxyapatite) is also common in obtaining scaffolds for TE using SLS 3D printing.

Among the most important parameters that may affect the final properties of the scaffold are the composition and properties of the powder (e.g., granulometry) and the laser point size. The SLS technique has excellent characteristics in terms of the control of the scaffold microstructure. However, the technique requires some post-processing for the removal of excess material. The availability of materials suitable for obtaining scaffolds for TE applications using the SLS process is also limited.

#### 4.1.4. Material Jetting (MJ) and Bending Jetting (BJ) 3D Printing

MJ and BJ printing operate in a similar way to standard inkjet printers. In MJ printers, the jet provided by multiple print heads is formed by a photopolymer solution that is subsequently cured by the UV source. BJ printing combines two feeding systems. The first one is identical to the dust feeding of the SLS technique. In contrast, the second one is the jet, which selectively supplies a binder, producing a local assembly at different points in each layer of polymeric powder, programmed according to a CAD model. Each new layer is incorporated over the previous one (which combines assembled zones with non-assembled zones). Once the 3D object is printed, the excess material is removed by suction and a final sinter is applied to fuse the material and provide mechanical integrity to the structure. The resolution and dimensional accuracy of MJ is the highest of the 3D printing technologies, however it is also the most expensive plastic 3D printing process and its application in TE is limited because of the narrow window of polymers available.

On the other hand, the resolution of the BJ technique is lower and depends to a large extent on the properties of the powder (particle size and flowability) and the binder (adhesion). However, BJ 3D printing is increasingly being used in TE, as the morphology of the scaffold can be precisely controlled (up to a scale of few µm) to mimic the structure and properties of the ECM of the tissue to be repaired or replaced. In addition, the operating costs associated with this technique are relatively low and their application prevents the use of cytotoxic organic solvents. In addition to polymeric materials, ceramic, metallic materials and a wide variety of composite materials can be printed by BJ 3D printing [49].

However, there are certain limitations that make the application of BJ 3D printing in TE a complex challenge. These include: (i) moderate availability of suitable materials (polymers and binders) for 3D printing; (ii) contractions and deformations (and even cracks) that may occur at the sintering stage, which may be partially overcome by slight corrections during the CAD modeling stage; (iii) The formation of small pores (<600 μm) make it difficult to eliminate the excess material; (iv) the use of high temperatures in the sintering stage can restrict the incorporation of specific biological components.

Despite all these drawbacks, BJ 3D printing is a technique with enormous potential in TE, the evolution of which is benefiting from the rapid advances that are currently taking place in printing techniques and in the development of new materials (polymeric, biopolymeric and composite materials) and their binders. Thus, it is worth mentioning the advances that are taking place in 3D bioprinting research, which have even led to the development of scaffolds that integrate cells and other biological agents in the structure, during the in situ manufacturing process of tissue constructs [107].

### 4.2. Current Trends

3D printing has shown its great potential to design biomaterials with multiscale, multimaterial and multifunctional architecture. It could easily realize the architecture design from macroscale to microscale and control the complicated composition of biomimetic objects [108]. However, the use of 3D printing in the design of advanced biomaterials for multiple tissue regeneration based on biomimetic strategy is still under development, needing more research before its use.

In a similar trend as the previous techniques described, acellular scaffolds were produced by 3D printing, in which cells were subsequently seeded. However, in the last years, most research is focused on the encapsulation of cells within the polymeric hydrogel-based scaffolds. This variant of 3D printing is called 3D bioprinting. This technique combines 3D printing (to recreate complex structures under digital control and with molecular precision) with cell culture (which remains embedded in the material that acts as a support). Thus, this technique opens the possibility of designing and creating biological scenarios as complex and specific cellular environments, which can be used in different fields of regenerative medicine. In this way, this technique tries to improve the incorporation of cells related to the tissue to be replaced to accelerate their regeneration, since with the conventionally used techniques, a homogeneous and efficient distribution of these cells in the biomaterial is not achieved. However, this technique is still under investigation since it presents some limitations such as the type of hydrogel used since natural hydrogels are generally weaker in mechanical properties, whereas the synthetic hydrogels are lacking in bioactive molecules or the lack of viscosity of collagen-based hydrogels, which should be improved via polymer crosslinking [109].

Thus, different printing technologies, such as inkjet-based bioprinting, stereolithography bioprinting and magnetic bioprinting, have been used, achieving the successful development of various complex tissues such as bone, cartilage, skin, heart and lung [103,110]. In this context, Rathan et al. investigated the use of scaffolds made up of a 3D printed PCL frame that has blinks with MSC, grow factors and a hydrogel. These scaffolds have a special high-strength, similar to cartilage, indicating a great potential for cartilage regeneration [111]. In a similar study, Bejleri et al. have manufactured a bioprinted cardiac patch consisting of cardiac progenitor cells, cardiac extracellular matrix hydrogel and gelatin methacrylate. This patch improves the angiogenetic capacity of patients while regenerating cardiac functionality [112]. Another trend that is gaining importance thanks to this technique is the incorporation of drugs into manufactured biomaterials. This technique allows the easy integration of drugs into the biomaterials in a homogeneous way, making the release of drugs optimal. In this context, Lai et al. developed a PLGA/TCP/Icariin scaffold by 3D printing for bone regeneration. The scaffold could achieve a controlled release of icariin, improving their effect in osteogenesis [113]. In addition, 3D printing can generate porous scaffolds with the same pore distribution, achieving reproducibility that other techniques do not allow [104]. On the other hand, there are other bioprinting technologies which are being currently developed. In this sense, techniques related with inkjet-based bioprinting such as micro-valve bioprinting or laser-assisted bioprinting can be highlighted among the reviews of Ng et al. (2017) and Kérourédan et al. (2018), respectively [114,115].

Nevertheless, although these techniques have generated great expectation in tissue engineering due to their wide range of possibilities, there are still many factors that are being analyzed today [103]. For this reason, several bioprinting-based techniques are being investigated like extrusion or vat polymerization. Ozbolat et al. (2016) proposed the current advances and future perspectives in extrusion bioprinting, whereas the study of Ng et al. (2020) described the process, materials used, and potential application of scaffolds produced via vat polymerization [116,117].

Furthermore, nowadays, 4D printing is a continuum of 3D printing technology that is now able to apply certain conditions or forms of stimulation such as temperature, pressure, humidity, pH, wind, or light [118]. In this way, the processability of different materials is being evaluated using this technique, as well as the influence of the processing conditions on the biomaterials generated [119]. In addition, various software programs are being developed to evaluate the tissue to be replaced, to create a 3D model that is as similar as possible, also building databases that allow the choice of the most suitable materials and processing conditions for it in each case [120]. Another interesting trend is related to the implementation of deep learning in the development of 3D bioprinted scaffolds, such as image-processing and segmentation, optimization and in-situ correction of printing parameters and lastly refinement of the tissue maturation process [121]. Thus, the biomaterial would specialize in the patient, improving their biocompatibility and adaptation.

## 5. Comparison and Future Perspectives

In this section, the future trends of the different processing techniques of scaffolds are compared and described. In this sense, as a summary, the advantages and disadvantages of each technique have been detailed in the following table (Table 2).

**Figure 6:**
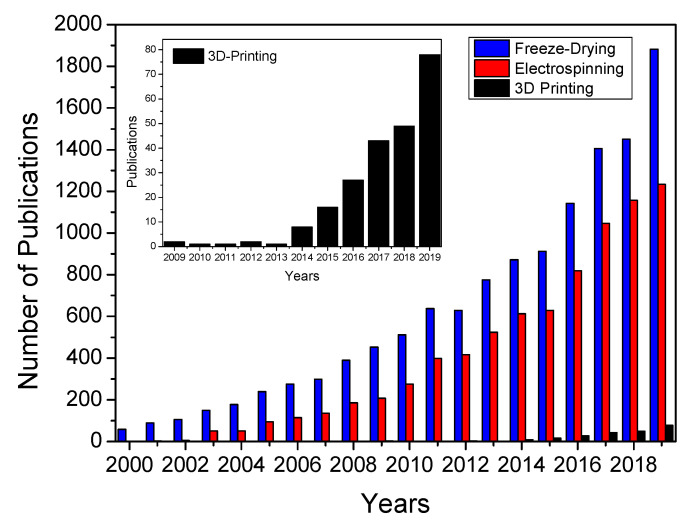
Evolution of the number of publications related to the use of freeze-drying, electrospinning and 3D printing in the biomaterials field. Data obtained from web of science. A magnification of the number of publications concerning 3D printing has also been included.

Figure 6 shows the number of publications concerning freeze-drying, electrospinning and 3D printing technologies related to the development of scaffolds since the year 2000. This type of graph is crucial to measure and compare the evolution of each technique over the years and its influence in biomedical sciences.

In respect to freeze-drying, an exponential growth is observed in the number of publications passing from 300 to more than 1400 in 2017 (blue bars). An increase over the years is observed, although this increase has been smoothed (comparing the publications of 2017, 2018 and 2019). On the other hand, the evolution of the number of publications concerning electrospinning also showed an exponential growth since 2006 (red bars). Interestingly, the impact of this technique over tissue engineering is also higher since a quarter of the publications concerning electrospinning are devoted to the development of scaffolds. According to the results, there is growing inertia based on the development of 3D electrospun scaffolds for soft-tissue engineering. To achieve this purpose, different approaches are being studied, e.g., modifying the electrospinning process to redesign the collector (to produce 3D structures instead of flat membranes scaffolds) or combining the electrospinning process with others as melt plotting or 3D printing. As for 3D printing (black bars), it is a novel and relatively recent processing technique of biomaterials, so no evolution is as clear as with previous techniques. However, its use is increasingly significant as it is verified in the research works carried out in this field in the last years (Figure 4). In this way, it can be predicted that this processing technique will be promoted in the next years by increasing its use exponentially due to its ability to obtain biomaterials with a specific and perfectly definable structure [131].

Another processing technique that is recently gaining interest in this field is the use of microfluidic-based foams, since it allows ease of control of the architecture of the scaffolds through the composition of the self-assembled liquid foams, thus improving their reproducibility and functionality [132,133]. This technique consists of the generation of monodisperse bubbles in a liquid that later solidify to form the 3D scaffold, allowing the achievement of regular and reproducible micro and nanometric pore sizes [134]. In this context, Lee et al. (2013) prepared a chitosan-based bioartificial liver chip using the same processing method [135]. On the other hand, Dalton et al. (2019) developed PEG shape memory scaffolds for vascular grafts using this technique [136].

In general, the polymeric materials with better biological character tend to have poor mechanical properties. Therefore, in most biomaterials and techniques, a field of interest (and which is gaining special relevance in the field of regenerative medicine) is related to coatings. In this way, a synergistic biomaterial can be produced with a material base with good mechanical properties and a specific coating that transmits optimal biological properties. Traditionally organic coatings have been used, mostly composed of polymers or copolymers that have different properties depending on their structure. In recent years, however, other types of coatings have become more relevant, the so-called hybrid coatings. These systems, made up of a mixture of organic and inorganic elements, make use of the advantages of both components to obtain materials with advanced properties in their interaction with biological systems. A clear example has been found in biomedical titanium alloys, since they have found a high resistance to corrosion, although in sliding and friction functions with other components, titanium suffers significant wear. Hence, different coatings that complement the properties of the biomaterial matrix have been sought and studied. This is where hybrid coatings come into play, since currently combinations are sought between materials that prevent wear (such as metals such as silver) and others that improve biocompatibility and cellular interaction (using natural antibodies such as collagen).

Definitively, the search for new materials and the use of existing ones to develop new applications is continuous, which is why tissue engineering is a field with great potential that can lead to systems with surprising properties for many different potential applications.

## Figures and Tables

**Table 1:** Summary of the effects produced by the most important variables of the electrospinning process on the morphology of the fibers formed.

Parameters		General Effects on the Morphology of Fiber Mats	References
**Composition parameters**	Concentration	Fiber diameter increases with the polymer concentration	[68,69]
		A decrease in concentration leads to formation of beads	
	Viscosity	There is direct relationship with concentration that leads to similar effects	[70,71]
		An increase in viscosity may prevent a stable flow through the nozzle	
	Molecular weight of polymer	Similar effect than viscosity that grows with increasing molecular weight	[72,73]
		An increase may prevent the occurrence of beads	
	Conductivity	An increase favors the formation of uniform fibers free of beads	[74,75,76]
		It also favors a reduction in size (with some exceptions)	
	Surface tension	There is not a general trend between surface tension and fiber morphology	[70]
	Volatility	A low volatility level may impair solvent removal	[77,78]
		A high volatility level may lead to ribbon-like and porous fibers	
	Dielectric constant of solvent	High values of the solvent dielectric constant favor electrospinning	[79,80]
		A secondary solvent may be added to increase the dielectric constant	
**Processing parameters**	Flow rate	Low flow rates give rise to small fiber diameters	[81,82]
		High flow rates may prevent full solvent removal before the target	
	Voltage	There is no clear correlation between voltage and diameter of fibers	[75,83,84]
		High voltage values may lead to formation of beads along the fibers	
		Very high voltages may lead to formation of ultrathin secondary filaments	
	Nozzle–collector distance	A minimum distance is required to produce solvent-free fibers	[85,86]
		Too long or too short distances may lead to formation of beads	
	Type of nozzle	Coaxial nozzles may be used to produce hollow fibers	[41,87]
		Multiple nozzles are used to increase the production scale of fiber mats	
	Collector	Metallic collectors lead to smooth fibers and porous collectors to porous fibers	[41,88]
		Rotatory drum collectors may be used to control fiber alignment in the mat	
**Environmental**	Temperature	A rise in temperature reduces the viscosity with its corresponding effects	[69,85,89]
		Tend to reduce fiber size and may lead to bead formation at high concentration	
		It may also extend the polymer concentration window for electrospinning	
	Relative humidity (RH)	Low RH values anticipate evaporation and solidification, increasing fiber size	[90,91,92]
		High RH causes water condensation on the filaments and polymer precipitates. This effect leads to thick and porous fibers, even preventing their formation.	

**Table 2:** Advantages and drawbacks of the different processing techniques to produce scaffolds.

**Conventional Technologies**	**Advantages**	**Drawbacks**	**References**
Freeze-Drying	High porosities (ca. 98%)	Small-scale and time-consuming production	[37,54]
	High interconnectivity of the porous network	High energy consumption	
	Channel-like pores and anisotropic structure	Use of cytotoxic organic solvents	
	Tunable pore size and structure	High sublimation time required	
	Capability of integrating bioactive molecules	Typical tissue shrinkage	
Electrospinning	Wide range of polymers (synthetic and natural)	Limitations to produce 3D scaffolds	[122,123]
	It produces continuous fiber on a micro-nano scale	Some of the solvents used can be cytotoxic	
	Control over fiber diameters and orientation	Poor control on pore size and shape	
	Versatile and well characterized technique		
**3D Printing technologies**	**Advantages**	**Drawbacks**	**References**
Stereolithography (SLA)	High resolution	Large number of monomers (resin) required	[124]
	Excess liquid can be relatively easily removed	Low range of materials for photopolymerization	
	Uniformity in pores and interconnectivity	A post-polymerization stage is typically required	
Fused Deposition Modeling (FDM)	High cost-effective processing	Limited to regular and simple porous structures	
	It allows the use of multiple nozzles	Low utility with non-thermoplastic polymers	[125,126]
	Suitable for the design and manufacture of scaffolds	Little application with biodegradable polymers	
	It allows deposition at moderate temperature		
Selective Laser Sintering (SLS)	Low operating cost	High operating temperatures are reached	[127,128]
	Excellent control of the scaffold microstructure	Complex removal of excess material	
	Suitable with ceramics, metals and composites	A post-sinter stage required	
Binder Jetting (BJ) 3D Printing	Relatively low operating cost	Small range of suitable polymers and binders	[129,130]
	It allows complex morphologies with good precision	Complex removal of excess material	
	Suitable for incorporating cells into the scaffold	Contractions and deformations of scaffolds	
		Post-processing stage at high temperature	
